# Liquid-based cytopathology test as a novel method to identify *Aspergillus* in patients with pulmonary aspergillosis

**DOI:** 10.1038/s41598-017-07564-3

**Published:** 2017-08-08

**Authors:** Yongchun Shen, Xiaorong Zhang, Wenyi Lin, Chun Wan, Qiyuan Li, Yong Jiang

**Affiliations:** 1 Department of Respiratory and Critical Care Medicine, West China Hospital of Sichuan University and Division of Pulmonary Diseases, State Key Laboratory of Biotherapy of China, Chengdu, 610041 China; 2 Department of Pathology, The First Affiliated Hospital of Jiujiang University, Jiujiang, 332005 China; 3 Department of Pathology, Chengdu Womens’ and Children’s Central Hospital, Chengdu, 610091 China; 4 Department of Pathology, West China Hospital of Sichuan University, Chengdu, 610041 China

## Abstract

Conventional cytopathology examination of respiratory samples can aid in identifying *Aspergillus* but with poor sensitivity, so this study aimed to assess the potential of the liquid-based cytopathology test (LCT) for improving the identification of *Aspergillus* in respiratory samples following Papanicolaou’s or Special staining with Grocott’s methenamine silver or periodic acid-Schiff staining. Paired bronchial brushing samples (n = 54) and sputum samples (n = 117) from 171 patients with pulmonary aspergillosis were prepared as slides using either conventional cytopathology or SurePath LCT. LCT slides were generally superior to conventional slides, showing smaller cell monolayer surface area, clearer background and more distinct stereoscopic cytological features. For Papanicolaou’s staining, LCT-prepared slides allowed a higher positive rate of *Aspergillus* identification than conventional slides for bronchial brushing samples (59.25% vs. 20.37%, *P* < 0.05) and sputum samples (29.05% vs. 8.55%, *P* < 0.05). Similarly, Special staining of LCT-prepared slides showed a higher positive rate of *Aspergillus* identification for bronchial brushing samples (83.33% vs. 57.41%, *P* < 0.05) and sputum samples (43.59% vs. 19.66%, *P* < 0.05). This preliminary study suggests that LCT may be better than conventional slide preparation for identifying *Aspergillus* in respiratory samples from patients with pulmonary aspergillosis.

## Introduction

*Aspergillus* is a ubiquitous fungus that causes a variety of clinical syndromes by interacting with the host^1, 2^. Pulmonary aspergillosis is caused mainly by *Aspergillus fumigatus* and has a spectrum of clinical syndromes, including invasive pulmonary aspergillosis (IPA), chronic pulmonary aspergillosis (CPA), allergic bronchopulmonary aspergillosis (ABPA), and aspergilloma^1, 2^. Pulmonary aspergillosis is a major cause of infectious mortality in immunocompromised patients, such as those receiving solid organ transplants or those with malignant disease or acquired immune deficiency syndrome, especially for patients with IPA^3^. Despite significant progress in understanding the pathogenesis of pulmonary aspergillosis and the availability of new fungicidal agents, as many as 30% of patients with IPA die from the disease around the world, and the mortality rate may be even higher among patients in critical care units^3, 4^. Such high mortality reflects the severity of comorbidities as well as delays in diagnosis, which in turn delay treatment initiation and compromise prognosis^5^. Diagnosis of pulmonary aspergillosis is often delayed due to low sensitivity and specificity^6, 7^. Thus, accelerating the diagnosis of pulmonary aspergillosis may significantly improve clinical outcomes.

Direct mycological examination under the microscope plays a central role in the diagnosis of pulmonary aspergillosis: cytopathological detection of *Aspergillus* in respiratory tract samples is essential for accurate diagnosis based on follow-up culture or histology examinations^6, 7^. In conventional cytopathological tests, a fresh tissue sample is placed on a slide, immersed in wet medium, stains are applied, and finally the slide is coverslipped. The stains may be Papanicolaou’s staining, or Special staining, usually Grocott’s methenamine silver (GMS) or periodic acid-Schiff (PAS) staining^7, 8^. Unfortunately, this conventional cytopathology shows poor sensitivity due to the presence of air-drying artifacts, overlapping clumps of cells and frequent presence of obscuring materials, such as necrotic tissue, mucus, inflammatory cells and blood^6, 7^.

A liquid-based cytopathological test (LCT), already commonly used to screen malignant cells in a variety of cancers, including cervical, thyroid, breast and lung cancers^9–13^, may provide benefits over the conventional cytopathological approach. LCT typically results in cleaner background and more evenly distributed cells, and LCT samples can be equally divided among multiple slides. Studies comparing LCT with traditional cytopathology indicate that LCT can show equivalent test accuracy and cytopathology features, while offering more satisfactory results^14, 15^. The present study aimed to compare LCT and conventional slide preparation techniques for identifying *Aspergillus* following Papanicolaou’s or Special staining of respiratory samples from patients with pulmonary aspergillosis.

## Patients and Methods

### Patient selection

This study was conducted according to the latest version of the Declaration of Helsinki, and ethical approval was obtained from the Ethics Committee of West China Hospital of Sichuan University. Upon admission, all patients in the study provided written informed consent for samples to be collected and analysed.

This study involved respiratory samples from 171 consecutive patients diagnosed with pulmonary aspergillosis in our hospital between March 1, 2012 and December 31, 2015. IPA patients were diagnosed with proven or probable based on the criteria of the European Organisation for Research and Treatment of Cancer/Mycoses Study Group (EORTC/MSG)^16^. CPA, ABPA and aspergilloma were diagnosed based on the expert consensus of the Chinese Thoracic Society and on guidelines of the European Society for Clinical Microbiology and Infectious Diseases as well as the European Respiratory Society^17, 18^. A total of 117 pairs of sputum samples were obtained from 117 patients, while 54 pairs of bronchial brushing samples were obtained from the remaining 54 patients.

### Papanicolaou’s staining

The 117 pairs of sputum specimens were divided into two sets; one was processed conventionally and the other was processed using LCT. For conventional processing, direct smears were prepared from the fresh samples, slides were fixed immediately in 95% alcohol and stained using Papanicolaou’s method. LCT processing was carried out using the SurePath LCT kit (Tripath PREP, BD SurePath, Burlington, NC, USA), one of the most commonly used LCT platforms^17, 18^. Samples were mixed with 10 mL of CytoRich^TM^ Red and 1 mL of mucolytic agent per 10 mL of sputum. The mixture was incubated at room temperature for 30 min, vortexed for 10 s, and centrifuged at 600 rpm for 10 min. The supernatant was removed and the pellet was resuspended in 10 mL of distilled water. This suspension was vortexed again and centrifuged at 600 rpm for 5 min. The supernatant was removed and the pellet was vortexed and transferred to the AutoCyte PREP system (Tripath Imaging) for automated slide preparation and staining.

The 54 pairs of bronchial brushing samples, obtained via fiberoptic bronchoscopy, were also divided into two sets: one was processed conventionally and the other was processed using LCT. Conventional preparation was as described for sputum samples. LCT preparation began with 30-min incubation of bronchial washing specimens in CytoClear^TM^ medium, followed by centrifugation at 600 rpm for 5 min. The supernatant was removed and the pellet was vortexed and transferred to the AutoCyte PREP system (Tripath Imaging) for automated slide preparation and staining.

### Special staining with GMA and PAS

Samples were prepared as described above using conventional or LCT procedures and stained with GMS or PAS instead of Papanicolaou’s stain. A Special Stains Automated Slide Stainer (NexEs, USA) was used with commercial kits (Roche Diagnostics, USA).

### Pathological examination

Two experienced cytopathologists blinded to patient status analysed the samples independently. Slides stained with Papanicolaou’s staining were considered positive if hyaline, septate mycelium filaments 2–4 μm in diameter with occasional branching at acute angles were observed. Slides stained with PAS were considered positive if magenta *Aspergillus* mycelium was observed; slides stained with GMS were defined as positive if brown-black *Aspergillus* mycelium was observed. Patients were defined as positive by Special staining only if both GMS- and PAS-stained samples were positive. Disagreements were resolved by discussion.

### Statistical analysis

Data were analysed using SPSS 18.0 for Windows (IBM, Chicago, IL, USA). Categorical data were expressed as absolute or relative frequencies, while continuous data were expressed as mean ± SD. Sensitivities of the conventional and LCT methods were compared using the χ^2^ test. The threshold of significance for all statistical tests was defined as a two-sided *P* < 0.05.

## Results

### Clinical characteristics of patients

The study involved 171 patients (age, 59 ± 13 yrs; male/female, 120/51) (Table 1). Duplicate sputum specimens were prepared from 117 patients, while duplicate bronchial brushing samples were prepared from the remaining 54 patients. Among the 117 patients with paired sputum samples, 63 had IPA, 39 CPA, 9 aspergilloma, and 6 ABPA. The corresponding numbers among the 54 patients with paired bronchial brushing samples were 25, 25, 4 and 0. Among IPA patients with paired sputum samples, 39 had proven disease while 24 had probable disease; all 25 IPA patients with paired bronchial brushings had proven disease. Substantial proportions of the 171 patients had serious comorbidities: 54 (31.59%) had chronic obstructive pulmonary disease, 38 (22.22%) had solid malignancies, 23 (13.45%) had diabetes, 20 (11.70%) had bronchiectasis, and 13 (7.60%) had tuberculosis. All patients were negative for human immunodeficiency virus (Table 1).

**Table 1:** Clinico-demographic data on patients.

	Patients with bronchial brushing samples	Patients with sputum samples
No. of patients	54	117
Sex		
Male	35	85
Female	19	32
Median age	55 ± 12	61 ± 14
Diagnostic tests		
Histopathology	54	81
Positive sputum culture	2	36
1, 3-beta-D-glucan assay (Positive/total)	3/15	36/102
Galactomannan assay (Positive/total)	0/7	0/10
Pulmonary aspergillosis		
IPA (Proven/Probable)	25(25/0)	63(39/24)
CPA	25	39
Aspergilloma	4	9
ABPA	0	6
HIV-positive	0	0
Underlying disease		
Solid tumor (Lung cancer)	22 (20)	16 (14)
Diabetes	4	20
COPD	4	50
Tuberculosis	1	12
Bronchiectasis	0	20

Values are N, or mean ± SD.

ABPA: Allergic bronchopulmonary aspergillosis; COPD: Chronic obstructive pulmonary disease; CPA: Chronic pulmonary aspergillosis; HIV: Human immunodeficiency virus; IPA: Invasive pulmonary aspergillosis.

### Cytomorphological characteristics of *Aspergillus*

**Figure 1:**
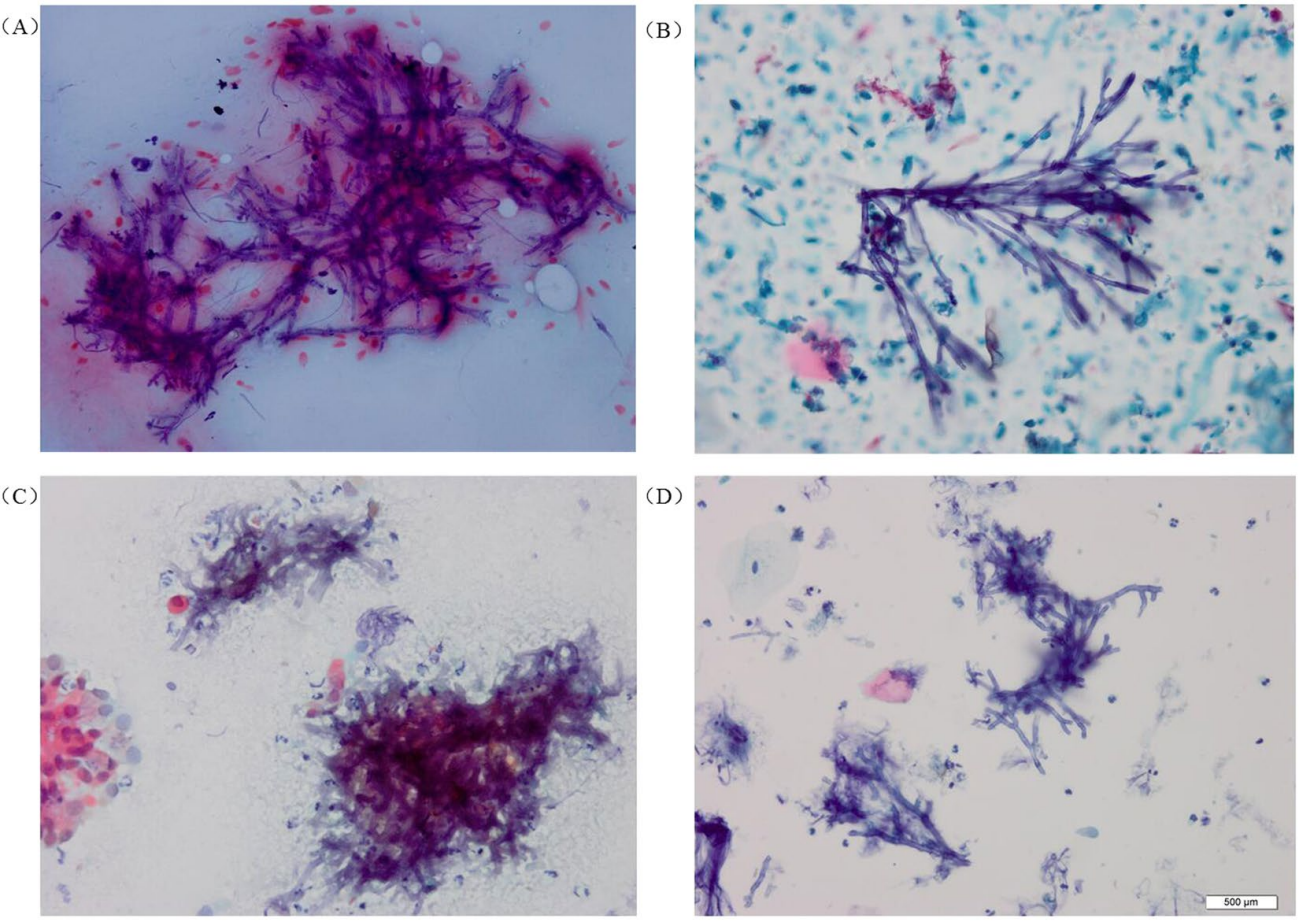
Cytomorphology of *Aspergillus* in sputum and bronchial brushing samples processed using the SurePath LCT platform or conventional cytopathology, followed by Papanicolaou’s staining. Typical cytological features of *Aspergillus* filaments included filament separation, constant filament diameter and parallel filament edges, with dichotomous branching at acute angles. Samples were stained with Papanicolaou’s stain. Magnification, 400X. (**A**) Conventional cytopathology on sputum samples; (**B**) LCT on sputum samples; (**C**) Conventional cytopathology on bronchial brushing samples; (**D**) LCT on bronchial brushing samples; LCT: Liquid-based cytopathology.

**Figure 2:**
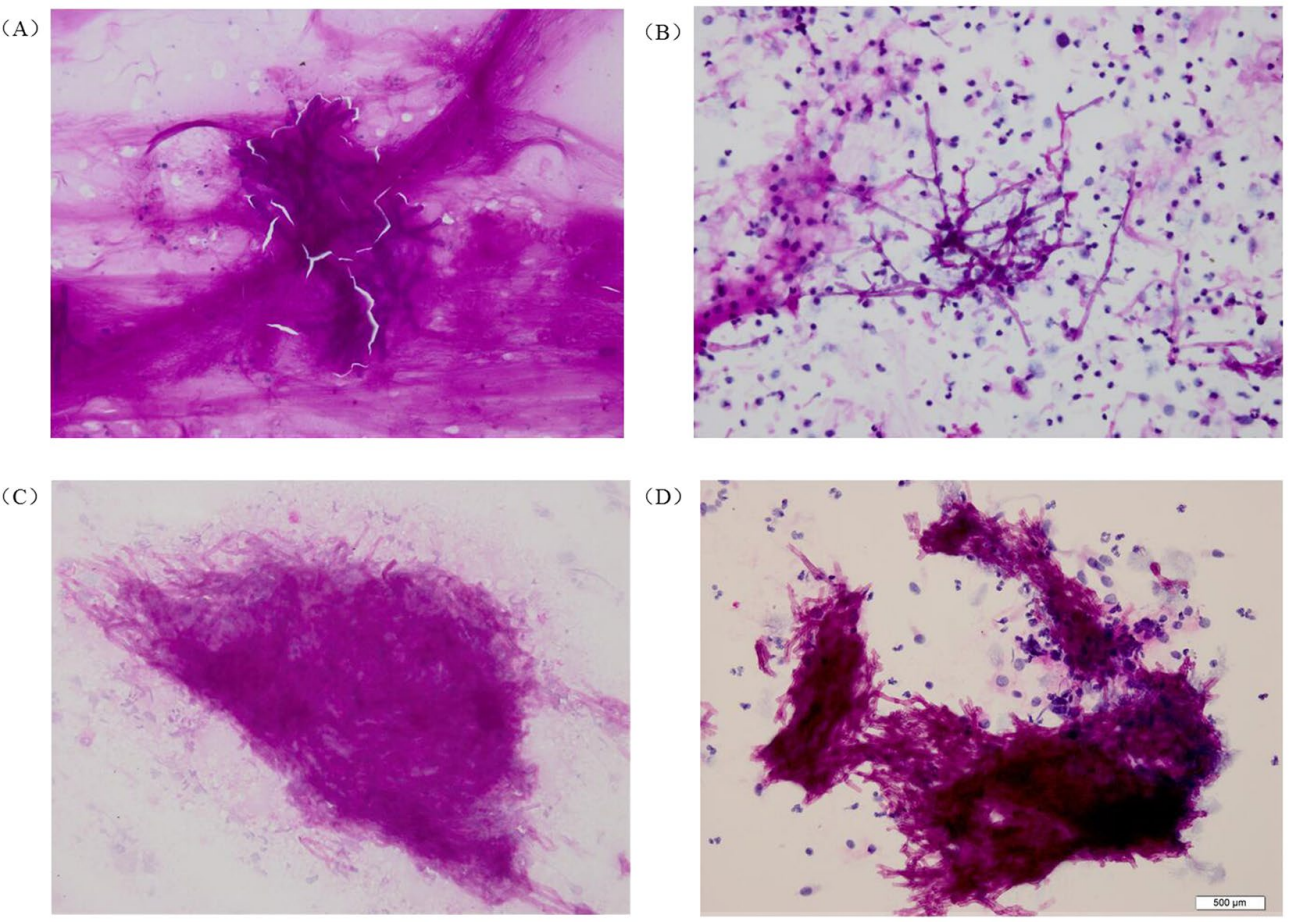
Cytomorphology of *Aspergillus* in sputum and bronchial brushing samples processed using the SurePath LCT platform or conventional cytopathology, followed by PAS staining. The mycelium of *Aspergillus* appeared magenta after PAS staining. Magnification, 400X. (**A**) Conventional PAS on sputum samples; (**B**) LCT-based PAS on sputum samples; (**C**) Conventional PAS on bronchial brushing samples; (**D**) LCT-based PAS on bronchial brushing samples. LCT: Liquid-based cytopathology; PAS: Periodic Acid-Schiff.

**Figure 3:**
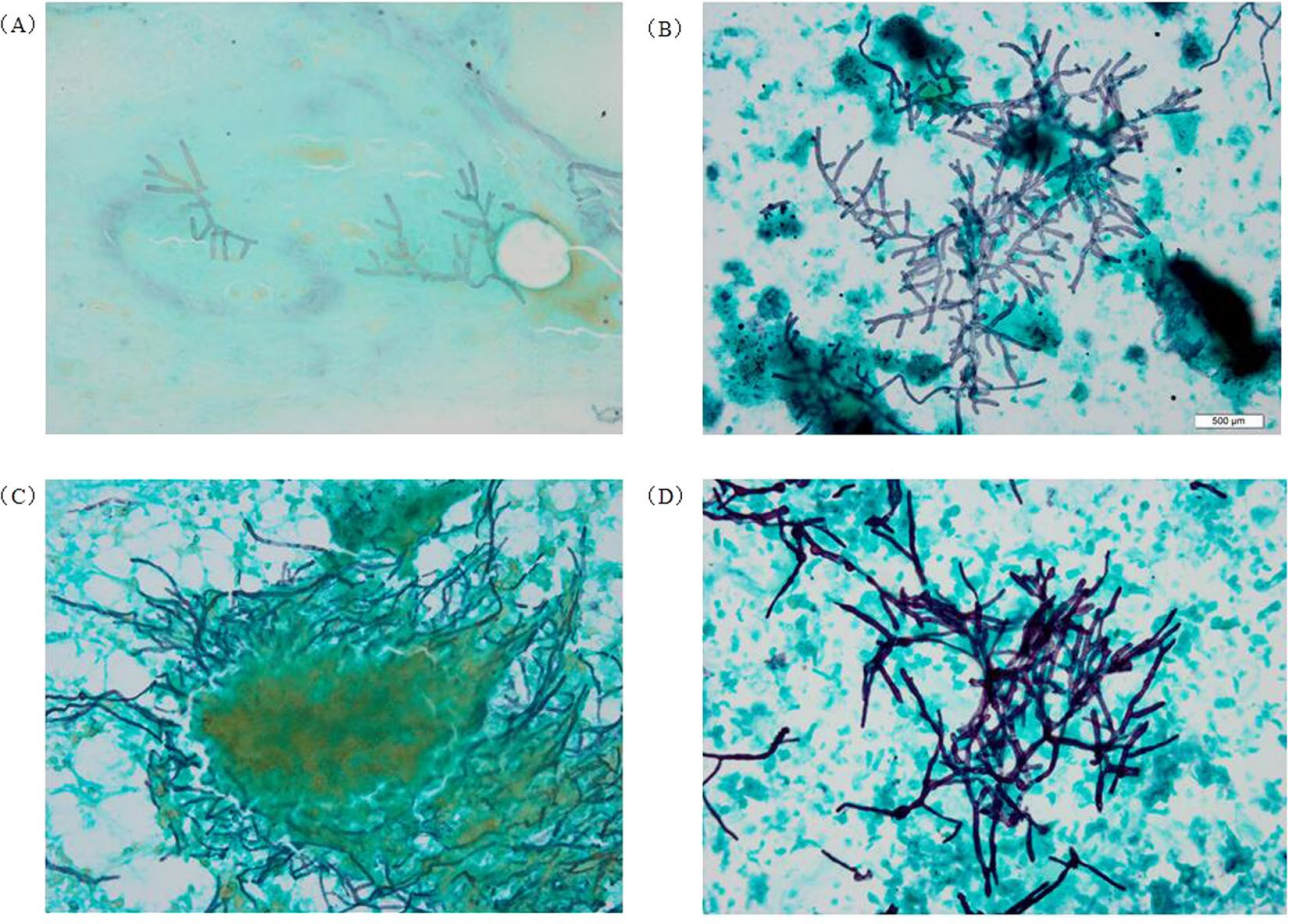
Cytomorphology of *Aspergillus* in bronchial brushing samples processed using the SurePath LCT platform or conventional cytopathology, followed by GMS staining. The mycelium of *Aspergillus* appeared brown-black after GMS staining. Magnification, 400X. (**A**) Conventional GMS on sputum samples; (**B**) LCT-based GMS on sputum samples; (**C**) Conventional GMS on bronchial brushing samples; (**D**) LCT-based GMS on bronchial brushing samples. LCT: Liquid-based cytopathology; GMS: Grocott’s Methenamine Silver.

The morphological characteristics of *Aspergillus* were generally clearer and more easily distinguished in LCT samples than in conventional samples. In sputum and bronchial brushing samples analysed by LCT with Papanicolaou’s staining, *Aspergillus* filaments showed a constant diameter, parallel edges, and dichotomous branching at acute angles (Fig. 1). The microscopic fields were generally clearer in LCT samples than in conventional ones, showing less necrosis, mucus, inflammatory cells and blood. Similar results were observed for LCT-prepared slides following Special staining (Figs 2 and 3).

**Figure 4:**
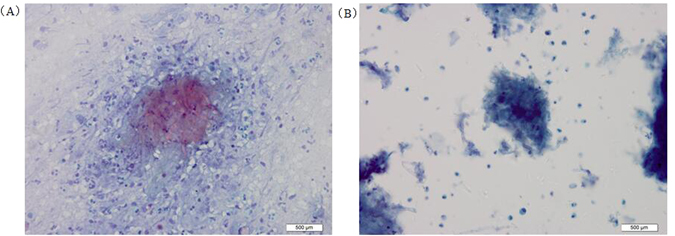
Cytomorphology of “branched ashes-like necrosis” in bronchial brushing samples processed using the SurePath LCT platform or conventional cytopathology, followed by Papanicolaou’s staining. Necrosis in the pattern of “branched ashes-like necrosis” appeared as wither-like ashes. Samples were stained with Papanicolaou’s stain. Magnification, 400X. (**A**) Conventional cytopathology on bronchial brushing samples; (**B**) LCT on bronchial brushing samples. LCT: Liquid-based cytopathology.

In Papanicolaou’s staining, a proportion of bronchial brushing samples showed distinctive cytopathology that we termed “necrosis-containing branch profile” or “branched ashes-like necrosis”. This feature was observed in 28 pairs of samples prepared conventionally and 10 pairs of samples prepared by LCT. All but one of the 10 paired samples that showed “necrosis-containing branch profile” after LCT also showed this result after conventional processing. This feature appeared under the microscope as patchy necrosis (mainly in LCT samples) or as lamellar necrosis (mainly in conventional samples), with light blue granular staining (Fig. 4). Areas of lamellar necrosis showed outlines of branching shapes with nearly the same size, like the ashes of burned branches, without tumor pyknosis or apoptosis.

### *Aspergillus* identification using Papanicolaou’s staining

The LCT platform led to identification of *Aspergillus* in 34 of 117 sputum samples, corresponding to a sensitivity of 29.05% (Table 2). This was significantly higher than conventional cytopathology (10 of 117, 8.55%, *P* < 0.001). The LCT platform led to identification of *Aspergillus* in 32 of 54 bronchial brushing samples, corresponding to a sensitivity of 59.25%. This was significantly higher than conventional cytopathology (11 of 54, 20.37%, *P* < 0.001).

**Table 2:** Results of conventional and liquid-based cytopathology.

Staining	Sample	Positive cases - conventional	Positive cases - LCT	P value
Papanicolaou’s	Bronchial brushing (n = 54)	11(20.37%)	32(59.26%)	<0.001
	Sputum (n = 117)	10(8.55%)	34(29.06%)	<0.001
Special staining	Bronchial brushing (n = 54)	31(57.41%)*	45(83.33%)*	0.003
	Sputum (n = 117)	23(19.66%)*	51(43.59%)*	<0.001

LCT: liquid-based cytopathology test.

*P < 0.05 *vs* Papanicolaou’s staining.

Next we investigated the performance of LCT in subgroups of patients with pulmonary aspergillosis. IPA patients showed the highest rate of positive *Aspergillus* identifications with LCT-prepared slides: 64.00% (16/25) in the case of bronchial brushing samples, 36.50% (23/63) in the case of sputum samples.

### *Aspergillus* identification using Special staining

Compared to Papanicolaou’s staining, Special staining with GMS and PAS led to much higher rates of positive *Aspergillus* identification in bronchial brushing samples prepared conventionally [20.37% (11/54) *vs* 57.41% (31/54)] or prepared using LCT [59.26% (32/54) *vs* 83.33% (45/54)] (both *P* < 0.05). Similar results were obtained with sputum samples (Table 2).

Comparison of conventional or LCT slide preparation followed by Special staining showed that LCT was associated with a higher rate of positive *Aspergillus* identification in bronchial brushing samples (83.33% *vs* 57.41%, *P* < 0.05) and sputum samples (43.59% *vs* 19.66%, *P* < 0.05). Among subgroups of patients with pulmonary aspergillosis, IPA patients showed the highest rates of positive identification (Table 3).

**Table 3:** Results of conventional and liquid-based cytopathology for diagnosing different types of pulmonary aspergillosis

	n	Papanicolaou’s staining		P value	Special staining		P value
		Conventional	LCT		Conventional	LCT	
**Bronchial brushing samples**	54						
IPA	25	7(28.00%)	16(64.00%)	0.011	16(64.00%)	23(92.00%)	0.017
CPA	25	4(16.00%)	13(52.00%)	0.007	12(48.00%)	18(72.00%)	0.083
Aspergilloma	4	0	3(75.00%)	0.028	3(75.00%)	4(100.00%)	0.295
ABPA	—						
**Sputum samples**	117						
IPA	63	7(11.11%)	23(36.51%)	0.001	17(26.98%)	38(60.32%)	<0.001
CPA	39	2(5.13%)	6(15.38%)	0.135	4(10.53%)	7(13.21%)	0.329
Aspergilloma	9	0	1(11.11%)	0.303	1(11.11%)	2(22.22%)	0.527
ABPA	6	1(16.67%)	4(66.67%)	0.079	1(16.67%)	4(66.67%)	0.079

IPA: invasive pulmonary aspergillosis; CPA: chronic pulmonary aspergillosis; ABPA: allergic bronchopulmonary aspergillosis.

## Discussion

Identification of *Aspergillus* from stained cytological slides plays an important role in the diagnosis and management of pulmonary aspergillosis, but conventional slide preparation has several disadvantages that make *Aspergillus* identification in respiratory samples challenging^19, 20^. In this study, we compared the ability of LCT and conventional cytopathology to allow *Aspergillus* identification in sputum and bronchial brushing samples following Papanicolaou’s or Special staining. We found that LCT allowed significantly better pathogen detection in both types of samples, suggesting that this method may help improve timely and accurate diagnosis of pulmonary aspergillosis.

To identify *Aspergillus*, conventional culture-based methods on respiratory tract samples show poor sensitivity of approximately 40% for diagnosing IPA and 17.9% for diagnosing CPA^21^. Meanwhile, biomarker tests are limited and there is no consensus about which sampling sites (blood, broncho-alveolar fluid or tissue biopsy) are more practical and result in more sensitive detection. For example, the serum galactomannan assay shows relatively low sensitivity in patients who have no haematological conditions or who are on antifungal therapy^22^. Tests based on the pan-fungal marker β-d-glucan do not discriminate between invasive aspergillosis and other invasive fungal infections^23–25^. EORTC guidelines do not include *Aspergillus* PCR as a mycological criterion for diagnosing probable IPA^16, 26^. Lung biopsy, which is considered a gold standard procedure, is highly invasive and available only at specialised institutions. These disadvantages of existing methods have helped drive efforts to develop new methods for rapidly identifying *Aspergillus* in order to facilitate early, accurate diagnosis of pulmonary aspergillosis.

Our results suggest that the LCT platform offers advantages over conventional cytopathology for both Papanicolaou’s and Special staining of sputum and bronchial brushing samples. Our results support the idea that LCT improves visualisation of cells: the cytopathology slides have uniform thickness; cellular structure is better preserved; and air-drying artifacts, obscuring blood and inflammatory exudate are absent. In addition, LCT-processed samples contain approximately 5-fold more sputum than conventional cytopathology samples, improving the chances of detecting *Aspergillus*. The fact that LCT slides are automatically prepared and stained in the AutoCyte PREP system helps ensure uniform quality of results, regardless of the practitioner’s experience. We found that the rate of positive identification with LCT-prepared slides was higher in bronchial brushing samples than in sputum samples (59.25% *vs* 29.05%), and that the positive rate on LCT-prepared slides was higher for IPA patients than for other subgroups with pulmonary aspergillosis. These results suggest that the diagnostic enhancement provided by LCT may be different for different tissues and subtypes of pulmonary aspergillosis. This should be taken into account when assessing the clinical utility of LCT.

Bronchial brushing samples from the lower respiratory tract can aid in the diagnosis of pulmonary aspergillosis^27^. Our analysis of 54 such samples revealed a potential novel type of pathological necrosis different from caseous necrosis or tumor necrosis: in some samples, when we observed necrosis and subsequently darkened the light source, we observed that the necrosis presented with wither-like ashes, which we named “necrosis-containing branch profile” or “branched ashes-like necrosis”. This level of cytomorphological detail into necrosis illustrates the greater clarity of subcellular structures in LCT samples than in conventional samples, since necrosis often masks *Aspergillus* in conventional smears. We suggest that “branched ashes-like necrosis” is a cytomorphological feature of *Aspergillus* in bronchial brushing samples, and that its detection can aid in the diagnosis of pulmonary aspergillosis. Specifically, “branched ashes-like necrosis” observed after Papanicolaou’s staining suggests the possibility of pulmonary aspergillosis, which should be investigated with further tests. Neutrophil karyopyknosis can also appear in cytological samples from patients with pulmonary aspergillosis, but such changes may reflect general diapyetic inflammation and are useful for differential diagnosis^28^.

Several studies recommend using Special staining such as PAS and GMS stain on all available samples, especially for immunodeficient patients^6–8^. We found that using Special staining increased the chance of identifying *Aspergillus* in sputum and bronchial brushing samples over Papanicolaou’s staining. We also found that positive *Aspergillus* identification rates were higher with LCT-prepared slides than with conventionally prepared ones. Our results suggest the benefit of using LCT with Papanicolaou’s or Special staining for the identification of *Aspergillus* in respiratory samples, which may improve diagnosis of pulmonary aspergillosis.

Taken together, our results suggest that LCT may be a novel method for identifying *Aspergillus* in respiratory samples. However, several considerations suggest that it cannot provide definitive diagnosis of pulmonary aspergillosis on its own. First, LCT cannot accurately type *Aspergillus* species, which may affect the selection of antifungal drugs. Second, international guidelines dictate that aspergillosis be confirmed through histology and isolation of *Aspergillus* from samples obtained through sterile procedures. Therefore, results of LCT analysis may provide information valuable for subsequent culture and invasive biopsy tests. Third, although LCT leads to higher rates of positive identification following Papanicolaou’s staining, the overall positive rate for sputum is still low (29.05%). This low sensitivity likely reflects that Papanicolaou’s stain is not specific^29^ and that it can give false negative results when small amounts of fungus are present, when sputum is of low quality or when the site of infection is not connected to the bronchus. Despite this low sensitivity, we suggest that Special staining of LCT-prepared slides should be performed on all available samples in order to maximise the possibility of identifying *Aspergillus*. In this study, LCT-based Special staining on sputum samples increased the positive rate to 43.59%, significantly higher than the rate with Papanicolaou’s staining (29.05%). Our results indicate that LCT-based Papanicolaou’s staining and Special staining can be useful for screening respiratory samples for the presence of *Aspergillus* in order to guide further biopsy and culture for definitive diagnosis. At the same time, this diagnostic approach may be useful in many critical ill patients who cannot tolerate invasive biopsy examination.

Our results with this small sample should be verified in larger, prospective diagnostic studies. The retrospective findings described here are based only on patients diagnosed with pulmonary aspergillosis, allowing us to calculate only the rate of positive identification (sensitivity) as a partial measure of diagnostic performance.

## Conclusion

This preliminary study suggests that LCT-based Papanicolaou’s staining and Special staining may be helpful for the identification of *Aspergillus* in sputum and bronchial brushing samples from patients with pulmonary aspergillosis. Our results justify further work, particularly prospective trials, to develop this assay for clinical use.

## References

[1] Kousha M, Tadi R, Soubani AO (2011). Pulmonary aspergillosis: a clinical review. Eur Respir Rev.

[2] Kosmidis C, Denning DW (2015). The clinical spectrum of pulmonary aspergillosis. Thorax.

[3] Cadena J, Thompson GR, Patterson TF (2016). Invasive Aspergillosis: Current Strategies for Diagnosis and Management. Infect Dis Clin North Am.

[4] Nivoix Y (2008). Factors associated with overall and attributable mortality in invasive aspergillosis. Clin Infect Dis.

[5] von Eiff M (1995). Pulmonary aspergillosis: early diagnosis improves survival. Respiration.

[6] Guinea J, Bouza E (2014). Current challenges in the microbiological diagnosis of invasive aspergillosis. Mycopathologia.

[7] Desoubeaux G, Bailly É, Chandenier J (2014). Diagnosis of invasive pulmonary aspergillosis: updates and recommendations. Med Mal Infect.

[8] Schelenz S (2015). British Society for Medical Mycology best practice recommendations for the diagnosis of serious fungal diseases. Lancet Infect Dis.

[9] Hoda RS, Loukeris K, Abdul-Karim FW (2013). Gynecologic cytology on conventional and liquid-based preparations: a comprehensive review of similarities and differences. Diagn Cytopathol.

[10] Kim SY (2016). Combined use of conventional smear and liquid-based preparation versus conventional smear for thyroid fine-needle aspiration. Endocrine.

[11] Michael CW, Bedrossian CC (2014). The implementation of liquid-based cytology for lung and pleural-based diseases. Acta Cytol.

[12] Gerhard R, Schmitt FC (2014). Liquid-based cytology in fine-needle aspiration of breast lesions: a review. Acta Cytol.

[13] Rossi ED, Bizzarro T, Longatto-Filho A, Gerhard R, Schmitt F (2015). The diagnostic and prognostic role of liquid-based cytology: are we ready to monitor therapy and resistance?. Expert Rev Anticancer Ther.

[14] Abedi-Ardekani B, Vielh P (2014). Is liquid-based cytology the magic bullet for performing molecular techniques?. Acta Cytol.

[15] Wu GP, Wang EH, Li JH, Fu ZM, Han S (2009). Clinical application of the liquid-based cytological test in cytological screening of sputum for the diagnosis of lung cancer. Respirology.

[16] De Pauw B (2008). Revised definitions of invasive fungal disease from the European Organization for Research and Treatment of Cancer/Invasive Fungal Infections Cooperative Group and the National Institute of Allergy and Infectious Diseases Mycoses Study Group (EORTC/MSG) Consensus Group. Clin Infect Dis.

[17] Infectious disease Committee of Chinese Thoracic Society (2007). Expert consensus on the diagnosis and treatment of pulmonary fungus diseases. Zhonghua Jie He He Hu Xi Za Zhi.

[18] Denning DW (2016). Chronic pulmonary aspergillosis: rationale and clinical guidelines for diagnosis and management. Eur Respir J.

[19] Rozemeijer K (2016). Comparing SurePath, ThinPrep, and conventional cytology as primary test method: SurePath is associated with increased CIN II+ detection rates. Cancer Causes Control.

[20] Qiu T (2015). Liquid-Based Cytology Preparation Can Improve Cytological Assessment of Endobronchial Ultrasound-Guided Transbronchial Needle Aspiration. Acta Cytol.

[21] Sherif R, Segal BH (2010). Pulmonary aspergillosis: clinical presentation, diagnostic tests, management and complications. Curr Opin Pulm Med.

[22] Von. Eiff. M (1995). Pulmonary aspergillosis: early diagnosis improves survival. Respiration.

[23] Kono Y (2013). The utility of galactomannan antigen in the bronchial washing and serum for diagnosing pulmonary aspergillosis. Respir Med.

[24] Pfeiffer CD, Fine JP, Safdar N (2006). Diagnosis of invasive aspergillosis using a galactomannan assay: a meta-analysis. Clin Infect Dis.

[25] Onishi A (2012). Diagnostic accuracy of serum 1,3-β-D-glucan for pneumocystis jiroveci pneumonia, invasive candidiasis, and invasive aspergillosis: systematic review and meta-analysis. J Clin Microbiol.

[26] Arvanitis M (2014). PCR in diagnosis of invasive aspergillosis: a meta-analysis of diagnostic performance. J Clin Microbiol.

[27] Verea-Hernando H, Martin-Egaña MT, Montero-Martinez C, Fontan-Bueso J (1989). Bronchoscopy findings in invasive pulmonary aspergillosis. Thorax.

[28] Coméra C (2007). Gliotoxin from Aspergillus fumigatus affects phagocytosis and the organization of the actin cytoskeleton by distinct signalling pathways in human neutrophils. Microbes Infect.

[29] Hettlich C, Küpper TH, Wehle K, Pfitzer P (1998). Aspergillus in the Papanicolaou stain: morphology, fluorescence and diagnostic feasibility. Cytopathology.

